# A pilot study of improved psychological distress with art therapy in patients with cancer undergoing chemotherapy

**DOI:** 10.1186/s12885-020-07380-5

**Published:** 2020-09-22

**Authors:** E. B. Elimimian, L. Elson, E. Stone, R. S. Butler, M. Doll, S. Roshon, C. Kondaki, A. Padgett, Z. A. Nahleh

**Affiliations:** 1 Department of Hematology/Oncology, Maroone Cancer Center, Cleveland Clinic - Florida, 2950 Cleveland Clinic Blvd, Weston, FL 33331 USA; 2 Department of Radiation Oncology, Dana-Farber Cancer Institute/ Brigham and Women’s Hospital, 75 Francis St, Boston, MA 02115 USA; 3 Charles E. Schmidt College of Medicine, Florida Atlantic University, 777 Glades Road BC-71, Boca Raton, FL 33431 USA

**Keywords:** Cancer, Intervention development, Art therapy, Survivorship care, Quality of life

## Abstract

**Background:**

Art therapy may improve the physical, mental, and emotional wellbeing of individuals for a variety of purposes. It remains understudied and underutilized in cancer care. We sought to determine the ability of a pilot art therapy program to improve the physical, mental, and emotional well-being of cancer patients.

**Methods:**

Chemotherapy-recipients, age 18 years and older, diagnosed with any type or stage of cancer, were considered eligible to participate in this single arm, pilot study, using four visual analog scales (VAS) with visually-similar, 0–10 scale (10 being worst) thermometers assessing: 1) pain, 2) emotional distress, 3) depression, and 4) anxiety. Participants were asked to complete all 4 metrics, pre-treatment, post-treatment, and at 48–72 h follow-up, after an hour-long art therapy session. Primary endpoints included post-intervention changes from baseline in the 4 VAS metrics.

**Results:**

Through a reasonable pilot sample (*n* = 50), 44% had breast cancer, 22% gastrointestinal cancers, 18% hematological malignancies, and 20% had other malignancies. A decrease in all VAS measures was noted immediately post-treatment but remained low only for pain and depression, not for emotional distress and anxiety upon follow up. There was a significant difference between the depression VAS scores of Hispanics (32%) compared to non-Hispanics (56%) (*p* = 0.009) at baseline. However, compared to non-Hispanics, Hispanics exhibited higher levels of depression after art therapy (*P* = 0.03) and during the follow-up intervals (*p* = 0.047).

**Conclusion:**

Art therapy improved the emotional distress, depression, anxiety and pain among all cancer patients, at all time points. While depression scores were higher pre-intervention for Hispanic patients, Hispanic patients were noted to derive a greater improvement in depression scores from art therapy over time, compared to non-Hispanics patients. Discovering simple, effective, therapeutic interventions, to aid in distress relief in cancer patients, is important for ensuring clinical efficacy of treatment and improved quality of life.

## Background

Cancer is a life-altering diagnosis. Surgery, radiation therapy, and chemotherapy can be effective in treating the tumor, but these modalities are not designed to treat the mental effects, which can be just as debilitating. The stress that results from a terminal illness, such as cancer, can be severe. The psychological distress associated with receiving a cancer diagnosis decreases quality of life and satisfaction with care; it can also impede medical compliance and increase health care costs [1–3]. The goals of cancer treatment include preventing recurrence, achieving remission or cure, prolonging life, and alleviating symptoms. Chemotherapy is a mainstay of cancer treatment but has several known side effects [4, 5]. Optimizing the quality of life of patients with cancer, during and after treatment, is an evolving field. Helping cancer patients strengthen their ability to cope with their diagnosis and improve their emotional and psychological well-being is important. Evidence exists to indicate that health outcomes are significantly improved when a patient has meaningful psychosocial support [6–10].

Complementary and integrative medicine programs have become key components of supportive care for cancer patients. The American Society of Clinical Oncology (ASCO) has recently endorsed the Society of Integrative Oncology (SIO) guidelines for Integrative Therapies during and after breast cancer [11]. These evidence-based guidelines recommend the use of integrative therapies for the management of symptoms and adverse effects, such as anxiety and stress [9]. Art therapy is among one of the recommended therapies proven to reduce anxiety and stress for breast cancer patients.

Art therapy can help cancer patients strengthen their coping mechanisms to achieve an improved quality of life [12]. However, it remains largely underutilized. Art therapy is a mental health service that employs the creative process of art-making to improve and enhance the physical, mental, and emotional well-being of individuals, of all ages, with a variety of diagnoses, including: chronic illness, physical disability, mental illness, and cancer [10, 13, 14]. It integrates the fields of human development (including visual art and harnessing the creative process through drawing, painting, sculpture, and other art forms) with models of counseling and psychotherapy [13–15]. It is based on the belief that the creative process involved in artistic self-expression helps patients resolve emotional conflict, develop interpersonal skills, manage behavior, reduce stress, increase self-esteem, enhance self-awareness, and achieve personal insight [13]. Art therapy has been reported to reduce depression and anxiety levels and aids in the overall improvement of quality of life for cancer patients [16–20] . However, there is still a paucity of data on the effects of art therapy on *distress* in patients affected by cancer [21] .

Over 35 years ago, Judith Rubin, a pioneering art therapist wrote “theory is only meaningful and worthwhile if it deals in a way that enables us to work better with [patients]. Theory and technique should [be] based on and growing out of the other, each constantly modifying the other over time” [22]. With theoretically-focused practice at the forefront for many art therapists, a push towards evidence-based art therapy practices requires increased accountability and transparency is being encouraged [21]. With this in mind, along with the SIO ASCO guidelines, we sought to implement an art therapy pilot study that would improve psychological distress and highlight and promote the voice of oncology patients to be incorporated in future art therapy programs.

The National Comprehensive Cancer Network (NCCN) defines distress as being, “along a continuum, ranging from common or normal feelings of vulnerability, sadness, and fears, to problems that can become disabling, such as depression, anxiety, pain, social isolation, and existential and spiritual crisis” [23]. The NCCN Distress Management Panel has recommended screening cancer patients for distress at initial and subsequent patient visits. Brief but effective screening instruments that can be readily available for a distress assessment led to the development of the distress thermometer, a 1-item distress screening tool [24]. Numerous studies have reported on the benefits of the distress thermometer in adult cancer patients [25–28]. Our institution routinely uses the NCCN “Distress Thermometer” to efficiently assess distress in our patient population and, as such, the Distress Thermometer is a tool our healthcare providers and oncology patients are familiar with in clinical practice.

Herein, we examine the ability of art therapy to improve the quality of life of cancer patients. Our pilot study seeks to demonstrate the clinical effectiveness of art therapy in decreasing the emotional burden of a cancer diagnosis, as captured by a visual analog scale (VAS) for emotional distress, depression, anxiety, and pain.

## Methods

### Study design and study population

We conducted a prospective, single-arm pilot study evaluating the effects of art therapy on anxiety, distress, and pain among cancer patients. Between April 2015 and February 2017, cancer patients receiving chemotherapy infusions or follow-up cancer care were recruited to participate in art therapy sessions. Patients meeting the following inclusion criteria were encouraged to participate in this pilot study: age of 18 years or older, diagnosis with any type or stage of cancer, actively undergoing chemotherapy at the Cleveland Clinic Maroone Cancer Center (Weston, FL), and the physical ability to attend and complete individual art therapy sessions (Eastern Cooperative Oncology Group Performance Status ≤1). All cancer types were invited to participate to determine if distress, anxiety, depression, pain and types of psychological stressors could be reduced regardless of cancer type and/or stage. All cancer types and stages were included as this study was exploratory in nature, in order to understand any benefit from implementing art therapy. Exclusion criteria included: any dementia, psychiatric illness, or cognitive/psychological limitation that might compromise the patient’s capacity to give informed consent.

Eligibility for study participation in this study was assessed by medical staff during patient clinical visits at the Cleveland Clinic Maroone Cancer Center in Weston, FL. If the patient was determined to be eligible, the physician presented information regarding the study. If a patient agreed to participate, they were referred to a research coordinator, who provided a more in-depth explanation of the study and obtained informed consent. All participants provided informed consent prior to enrollment. Eligible, consented participants, were enrolled in this study. Only data from patients who chose to participate in the study are presented in this report and used to determine the effectiveness of the art therapy sessions. All participants self-identified as being able to read and speak English.

### Study tools

To determine the psychosocial impact of the art therapy sessions, the Universal Pain Assessment Tool and the NCCN Distress Management Screening tool (version 1.2012) were modified and adapted to measure distress and mood in the form of a VAS. The reliability of the Universal Pain Assessment Tool and the NCCN Distress Management Screening tool in demonstrating efficacious, self-reported outcome measures is well established in the literature; both tools are widely used [24, 29]. Additionally, the NCCN Distress Thermometer, has been shown to be effective in assessing distress among oncology patients, and provides an accurate reflection of mood, depression, and anxiety [30, 31]. Study participants were encouraged to complete the demographic questionnaires and the VAS at various time points of the study.

Demographic questionnaires were completed at baseline. Within the demographic questionnaires, patients were also asked to identify personal or social influences that might contribute to their distress, depression, anxiety, and pain through a problem checklist consisting of nine primary concerns/psychosocial stressors: 1) financial concern, 2) family/caregiver concern, 3) job concern, 4) health concern, 5) relationship concern, 6) existential concern, 7) treatment concern, 8) identity concern, and 9) other. The demographic questionnaires allowed the authors to explore differences in response to the art therapy session by ethnicity, race, age, gender, marital status, and patient perceived causes of distress, anxiety, and depression.

**Fig. 1:**
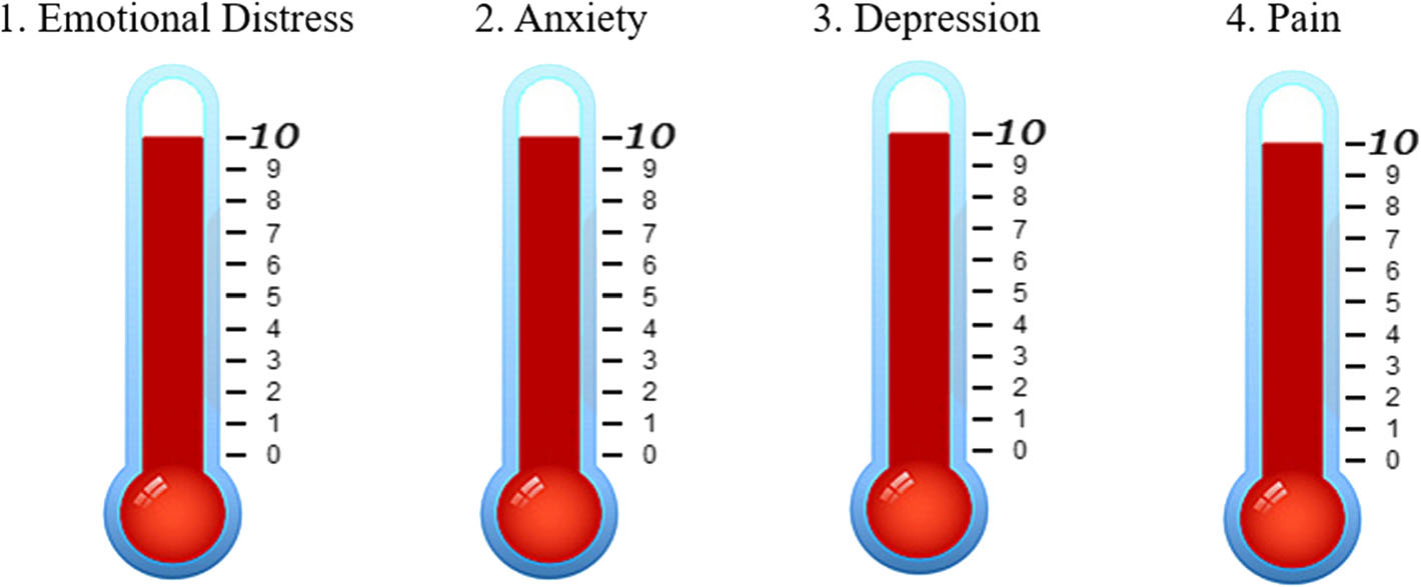
Four thermometers testing (0–10 scale) emotional distress, pain, depression and anxiety experienced within the past week, with 0 being the least amount of upset/pain and 10 being the highest amount

The four VAS thermometer assessments were administered on three separate occasions: pre-art therapy session, post-art therapy (immediately after the session), and within 48–72 h after the art-therapy session (by phone with the art therapist). Altogether, patients were administered four, visually similar thermometers: 1) a Pain Assessment Thermometer, 2) an NCCN-based Emotional Distress Thermometer, 3) a Depression Thermometer, and 4) an Anxiety Thermometer (Fig. 1). Patients were asked to mark the number (0–10), on each thermometer, which best described their state of emotional distress, depression, anxiety, and pain. Scoring was based on an ascending scale of severity: 0 being no perceived distress, depression, anxiety, or pain; 10 being the worst possible distress, depression, anxiety, or pain. Participants were asked to complete the same four VAS thermometers immediately prior-to, and after, the art therapy session.

### Art therapy sessions

The art therapy sessions were led by the same art therapist, at the Cleveland Clinic Florida Maroone Cancer Center. This registered therapist completed a master’s-level, graduate education in art therapy and completed post-education supervised clinical experience. The art therapist had previous experience working in the field of oncology and comprehensive knowledge of the theories and clinical skills used in art therapy for this patient group. Each art therapy session encouraged the patient to engage their creative side and employed consistent, standardized art therapy practices aimed to engage the mind, body, and spirit of the participant. Each participant was allowed full freedom of materials to work with, including supplies for painting, drawing, clay work, and collage construction. Sessions concluded with an art therapist-guided patient reflection on their own art.

### Data collection

Data collected included demographics, cancer stage, and time between cancer diagnosis and study enrollment. Study data was collected and managed using REDCap (Research Electronic Data Capture), hosted at the Maroone Cancer Center. The REDCap software is a secure web-based data collection tool, designed to support data capture for research studies.

### Statistical considerations

This was a single-center, single-arm, non-controlled prospective pilot study. The primary endpoint of the trial was to evaluate the psychosocial therapeutic effects of one art therapy session on patient distress, depression, anxiety, and pain, using four VAS assessments. The VAS assessments were administered in-person, both prior-to (baseline) and immediately after the art therapy session, as well as by phone 48–72 h after the art therapy session. Therapeutic effects were evaluated as any change, between intervals, for each VAS assessment. Sample size calculations indicated a sample of 50 patients would allow a detection of a change in scores > 2.5 points with > 80% power and α < 0.05.

Normality testing was executed with Kolmogorov-Smirnov (KS) analyses (verified with Normal Q-Q plots), to determine the most appropriate paired testing method to suit the distribution characteristics of this data set.

A secondary goal was to utilize multivariable regression analyses to assess any association between trends in VAS scores and the independent demographic characteristics of our patient population. The independent variables of interest for multivariable analysis were race (white vs. non-white), ethnicity (Hispanic vs Non-Hispanic), marital status (married vs. not married), age (< 50 years, 50–69 years, ≥70 years), and gender (female vs. male). This modeling method was used for each VAS assessment.

All significance was determined by a threshold of α < 0.05. All statistics were performed using SPSS software (SPSS Statistics, Version 25.0: IBM Corp., Armonk, NY).

### Ethical considerations

Study data was collected as a component of clinical care. Additional information analyzed from patient records was blinded and strictly controlled to ensure the patients’ privacy rights. Approval for data review and analysis was granted by the Cleveland Clinic Institutional Review Board. Clinical trial information: NCT02659345.

## Results

**Fig. 2:**
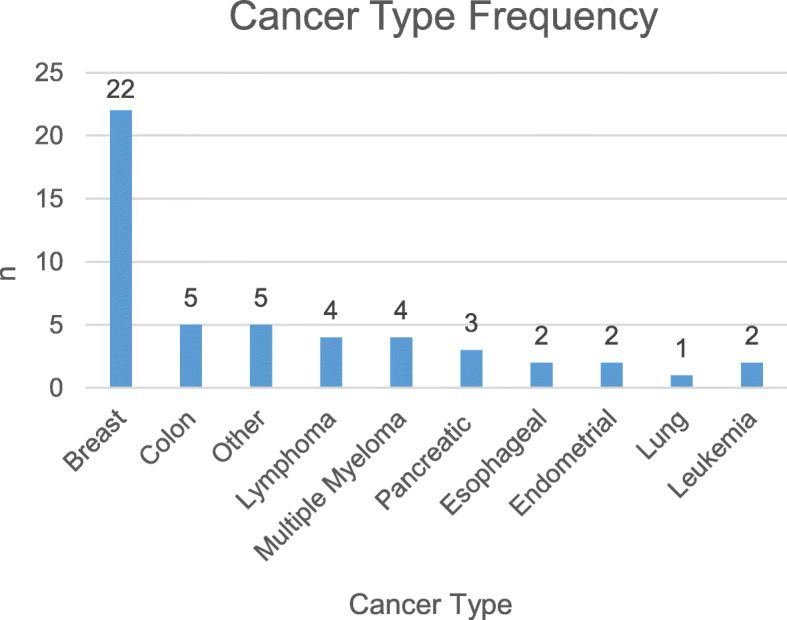
Observed frequency of cancer types within patient cohort

Fifty-five patients were enrolled into this prospective cohort study. Four patients failed to complete all assessments, and one patient passed away prior to study completion. Thus, the following results are reported on 50 patients who completed all questionnaires and VAS assessments, and who participated in the study in its entirety. The average age of our study participants was 54 years (range 42–66); 41(82%) were female and 9(18%) were male; 19(38%) were unmarried and 31(62%) were married; self-identified race included: 32(64%) white patients, 14(28%) black patients, and 4(8%) who identified as being “other”; with respect to self-identified ethnicity, 16(32%) were Hispanic, 28 (56%) were non-Hispanic, and 6 (12%) identified themselves as “Caribbean”, which the authors included into the “non-Hispanic” category. The majority of our patients (76%) were enrolled less than 1 year after receiving their cancer diagnosis; 5 (10%) were 1–3 years since first being diagnosed with cancer and 7 (14%) were more than 3 years after diagnosis. This tabulated demographic data can be found in Table 1. Patient cancer types included: breast (44%), pancreatic (6%), esophageal (4%), lung (2%), colon (10%), endometrial (4%), leukemia (4%), lymphoma (8%), multiple myeloma (8%), and other (10%) (Fig. 2). Cancer stages included: stage I (29%), stage II (27%), stage III (32%), and stage IV (12%).

**Table 1:** Patient Characteristics at Presentation

Marital Status	Number	%	***P***-Value (*denotes significance)
Married	31	62	0.0164*
Single, widowed, or divorced	19	38	
Total	50	100	
**Gender**			
Male	9	18	< 0.001*
Female	41	82	
Total	50	100	
**Age (mean +/− SD)**	**54.3 +/− 12.3**		
18–49	19	38	
50–69	28	56	< 0.001*
70+	3	6	
Total	50	100	
**Race**			
White	32	64	< 0.001*
Black	14	28	
Other	2	4	
More than 1 race	2	4	
Total	50	100	
**Ethnicity**			
Hispanic	16	32	
Non-Hispanic	28	56	< 0.001*
Other	6	12	
Total	50	100	
**Time Since Diagnosis** ^**a**^			
< 1 year	38	76	
1–3 years	5	10	< 0.001*
> 3 years	7	14	
Total	50	100	
**Type of Cancer**			
Breast Cancer	22	44	< 0.001*
Colon Cancer	5	10	
Endometrial Cancer	2	4	
Pancreatic Cancer	3	6	
Esophageal Cancer	2	6	
Multiple Myeloma	4	8	
Lymphoma	4	8	
Leukemia	2	2	
Lung	1	2	
Other ^b^	5	10	
Total	50	100	

^a^. Year since diagnosis is time between diagnosis/earliest note with cancer diagnosis and enrollment

^b^. Other Cancer Types Includes: Cervical, Uterine, Myelodysplastic Syndrome (MDS), Peritoneal Cancer

**Fig. 3:**
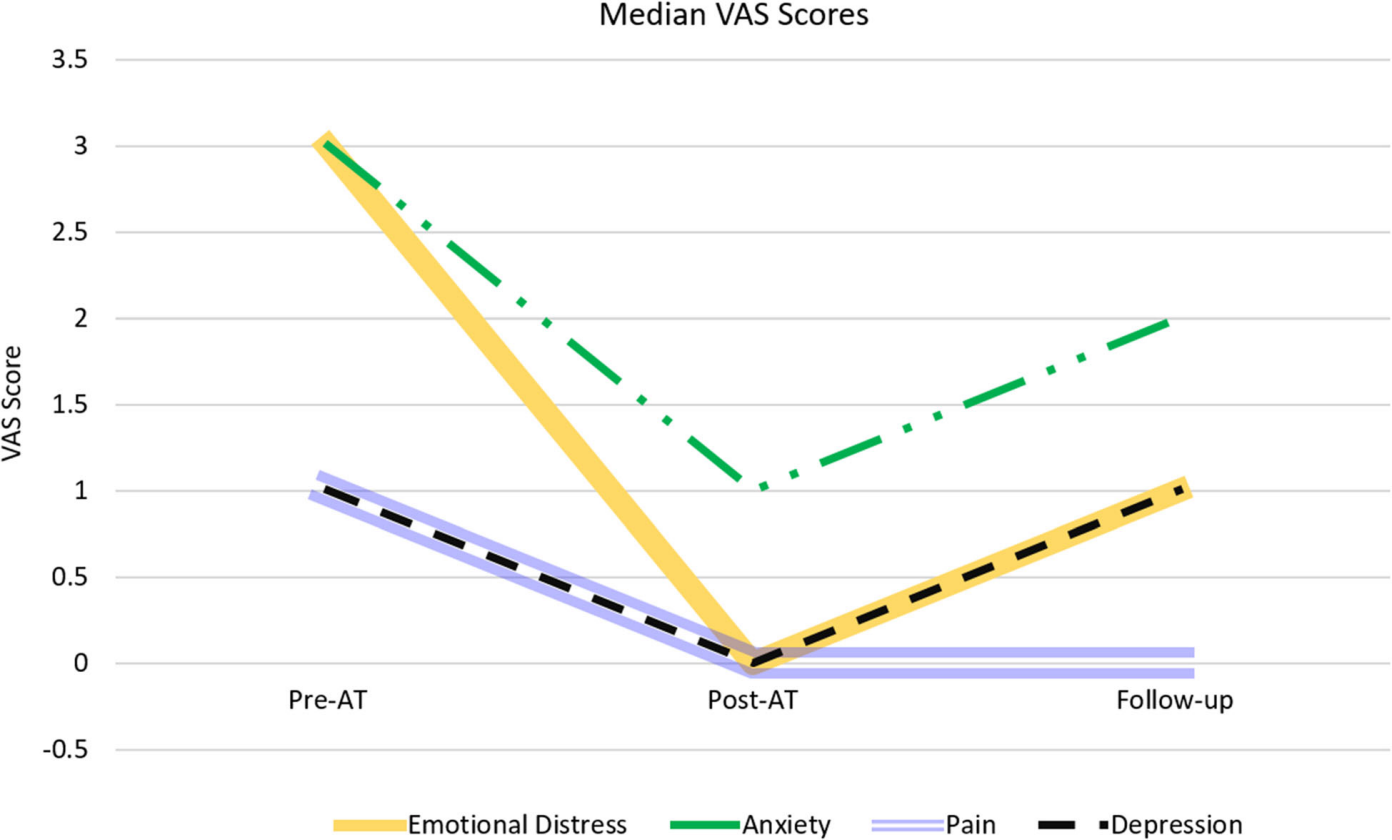
Median VAS scores (cohort overall) observed during each interval

Testing for normality with KS analyses indicated that all VAS outcomes data was non-normally distributed (*p* < 0.001, all VAS scores); these results were verified with normal Q-Q plots which confirmed non-normality. Therefore, Wilcoxon signed rank tests were executed to determine any significant changes in paired scores, between pre- and post-art session, as well as between post-art therapy session and follow-up. There was a statistically significant reduction, in all median VAS scores, between pre-art therapy session and post-art therapy session (*p* < 0.001, all VAS) (Table 2, Fig. 3). There were no significant differences between post-art therapy session and with follow-up for the pain and depression VAS scores, indicating continuous benefit in follow-up. There were however, significant differences noted between emotional distress (*p* = 0.023) and anxiety VAS scores (*p* < 0.001) which increased again at follow-up after decreasing immediately after post-art therapy session (Table 2, Fig. 3).

**Table 2:** Paired VAS Score Comparisons

	Pre-Session Median (Range)	Post-Session Median (Range)	*P* - value (*significance)	Post-Session Median (Range)	Follow-up Median (Range)	*P* - value (*significance)
Emotional Distress	3 (0-10)	0 (0-8)	< 0.001*	0 (0-8)	1 (0-7)	0.023*
Pain	1 (0-8)	0 (0-7)	< 0.001*	0 (0-7)	0 (0-7)	0.07
Depression	1 (0-8)	0 (0-5)	< 0.001*	0 (0-5)	1 (0-5)	0.16
Anxiety	3 (0-10)	1 (0-5)	< 0.001*	1 (0-5)	2 (0-5)	< 0.001*

Backwards multivariable regression, at baseline, revealed that there was a significant difference between the depression VAS scores of Hispanics compared to non-Hispanics (*p* = 0.009), with Hispanics exhibiting higher levels of measured depression when enrolled. This significant trend was also observed after the administration of art therapy, with Hispanics exhibiting higher levels of measured depression post-art therapy (*p* = 0.030) and at the follow-up intervals (*p* = 0.047). Also of note, while the Hispanic group did exhibit higher median levels of depression at all intervals, paradoxically, this group also exhibited a larger decrease in median depression, immediately following art therapy, than the Non-Hispanic group (a decrease of 3 points vs. 1 point on the VAS depression scale, for Hispanics and non-Hispanics, respectively). There were no other significant contributing patient characteristics observed in the regression models, for all VAS scores, at all intervals.

**Fig. 4:**
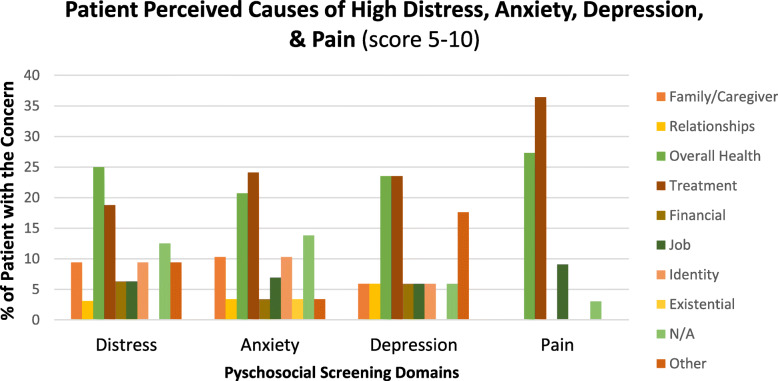
Patient Perceived Causes of High Distress, Anxiety, Depression, and Pain. High distressed was classified as a score of 5–10 on VAS for distress, anxiety, depression and pain

A high degree of emotional distress, anxiety, depression, or pain was defined as a score of 5–10 on the four, respective VAS assessments. Analysis of patient-perceived causes of high emotional distress, anxiety, depression, and pain indicated that the patient’s overall health and medical treatment was of increased concern across all VAS (Fig. 4). Of the patients with a high distress score, 8 (25%) were primarily concerned about their overall health and 6 (18.8%) were concerned about their medical treatment. Of the patients with a high anxiety score, 6 (21%) were concerned with their overall health and 7 (24%) were concerned with their medical treatment. Of the patients with a high depression score, 4 (24%) were equally concerned with their overall health as they were with their treatment. Of the patients with a high pain score, 3 (27%) were concerned with their overall health and 4 (37%) were concerned with their medical treatment.

## Discussion

This pilot study analyzed the effect of art therapy on the distress, anxiety, pain, and depression levels in patients with cancer. Our results suggest that art therapy effectively improved mood, and reduced both anxiety and pain levels among all patients with cancer receiving chemotherapy. Benefits were seen immediately after the art therapy session and persisted for both pain and depression at least 48–72 h afterwards, across all groups. Emotional distress and anxiety also improved immediately after art therapy, but the effect was short-lived and was not sustained on follow-up. Despite the temporary effects, the improvement in emotional distress is promising. The trend noted in our pilot study, indicating a decrease in self-reported distress levels with participation in our art therapy sessions, is encouraging and emphasizes the need for future research.

These findings are consistent with other studies reporting the benefit of art therapy in cancer, [32] on emotions in women with breast cancer [33], and as a form of mindfulness that can reduce the amount of stress, anxiety, and depression among patients with breast cancer [34–36] and those undergoing palliative care for cancer [37]. Several recent studies have also explored the effects of art therapy on quality of life in patients with cancer while either receiving radiotherapy [38, 39], or chemotherapy [40, 41]. Art therapy has been noted to provide patients with cancer the opportunity for self-development and optimistic reflection on life [42], and as a means to relax, develop a self-narrative, or visually express and elaborate on complex emotions [43]. Specifically, significant improvements in both depression and anxiety scores have been reported in patients with cancer when creative psychological interventions, like art therapy, are utilized [44]. Our data is also consistent with previously-reported studies which have indicated that art therapy reduces the global distress among cancer patients, with a significant reduction in all symptoms studied, including pain, anxiety, ill-being, tiredness, sadness, and depression [45]. Our findings also suggest that art therapy is likely to be beneficial when administered repeatedly and frequently, as the benefit was evident for all measures immediately post-session, but psychosocial effects were short-lasting for distress and anxiety.

Our data suggests that Mandala drawings and coloring, the primary art modalities utilized for art therapy, are beneficial. The implementation of Mandala art aims to reach individuals at their center and essence; encouraging people to be sincere with themselves [46]. Mandala art therapy is being utilized more frequently in psychiatric inpatient units, in an attempt to help patients merge their different life components [47]. All patients who chose Mandala art as their art therapy modality, found it to be beneficial in this study. Further research regarding which specific art therapy modalities might be of the greatest benefit is needed.

One strength of our study is that it evaluated the effect of several forms of art therapy on distress of patients with cancer, regardless of cancer type. Distress is a significant emotional upset stemming from a single or combined physical and psychiatric conditions [48]. Identification and measurement of patient psychological state of distress, utilizing a simple, cost-effective tool that can assess discrete levels of anxiety, depression, and/or pain, is essential for proper care and management. Several screening tools have already been described as effective, reliable, and feasible methods of detecting distress and the psychosocial needs of a patient [49–51].

Another unique strength of our study is proposing the utilization of a modified 1-item distress thermometer and a VAS as a convenient and potentially widely-applicable tool to screen, identify, and measure distress based on anxiety, depression, and pain. Our tool was adapted based on the commonly used NCCN Distress Thermometer already employed in the field of oncology. Approximately 40 standardized instruments have been used to quantify psychological distress in patients with cancer, and 7 are most commonly used [52]. These instruments share the common characteristics of being relatively brief but are not specific to patients with cancer. Despite the importance of the importance of assessing distress in oncology, several providers either do not routinely address distress, do not use screening instruments in their assessment, or do not feel they have adequate training in addressing psychosocial issues [53]. Also, many feel that some of the standardized tools, like the 14-item Hospital Anxiety Depression scale (HADS), that can be used to measure anxiety and depression are time consuming and may be difficult to administer in some clinical environments [25]. A study of 300 oncology professionals found the ideal distress screening tool to be short (1–3 questions in length) or an abbreviated version of a previously-validated questionnaire [53]. Once distress was identified, the study found that the majority of nurses (90%) versus physicians (40%) felt prepared to provide patients under distress the time and attention they needed. The clinician’s willingness to use advanced screening methods was dependent on the time clinicians were ready to spend identifying distress. The distress thermometer was found acceptable to ~ 75% of the clinicians surveyed. Based on this, we believe the simple instrument used in this study may be a useful and convenient tool to encourage busy clinicians to assess distress in patients with cancer more frequently.

Our results also suggest another potential application of the modified distress thermometer in helping providers to further assess and determine which patients might readily adapt and adjust to having a cancer diagnosis versus those needing additional support. Our analysis indicated that factors that were most concerning for patients with a high distress score were primarily concern about their overall health (25%) and concern about their medical treatment (18.8%). Similarly, those with a high anxiety, depression, or pain score, also identified their overall health and medical treatment as their primary concern. This is consistent with previous research suggesting that an individual’s ability to adapt to having a cancer diagnosis is significantly based on pre-existing psychosocial factors [54]. Further research is needed to confirm what psychological factors drive higher distress scores in patients with cancer and help inform future social support services that might be beneficial to cancer patients.

Our analysis revealed that this tool, being primarily based on a visual scoring system, could be potentially applicable across several ethnic and cultural groups with different language preferences. Interestingly, while Hispanic patients had higher depression scores at baseline, they exhibited the largest overall reduction in their depression when compared with Non-Hispanics at post-session, and follow-up intervals, suggesting a greater benefit from the art therapy intervention despite higher depression scores. Our findings, noting a significant difference between baseline depression scores of Hispanic patients versus non-Hispanic patients, is also supported in the literature. In the Unites States, studies on depression in non-Hispanic Whites and ethnic minorities reveal that Hispanics tend to report higher levels of depression overall compared to non-Hispanic Whites [55–58]. Additionally, studies utilizing the Center for Epidemiologic Study Depression Scale (CES-D) in measuring depressive symptoms among minority groups and White Americans reported that 15% of Non-Hispanic Whites and 26% of African Americans scored above the cut-off for depression compared to 32% of Cuban Hispanics and 30% of Non-Cuban Hispanics [56]. Moreover, it has been reported that older Hispanics, especially the elderly, face difficulties in recognizing and adequately treating their depression [59]. Increased levels of depression among ethnic minorities including the Hispanic populations, the largest minority group in the United States and the fastest growing minority group overall, is particularly concerning [60, 61]. While there exists cultural variations in the perception of depression and various culture-specific symptoms used to describe depression [62], this study suggests that simple interventions, based on art therapy, might be beneficial to improve distress and depression in cancer patients among various races, including Hispanics. Art therapy might, therefore overcome language and cultural barriers for all patients, including minorities, as it has been found to be suitable for patients with various diagnoses, ages, and levels of education [63].

Limitations of this study include the small sample size, which was, however, deemed statistically appropriate for this pilot study. A larger study should be performed to confirm these results, particularly the preferential benefit seen in Hispanics compared to non-Hispanics. Additionally, further tools could be used for a more in-depth analysis of the sources of distress, anxiety, depression, and pain. For example, future studies can employ the use of our modified VAS for depression in conjunction with specific depression scales such as the Beck Depression Scale to allow for further quantification of the degree of depression and to measure specific characteristics, or to validate the VAS scales utilized [64].

## Conclusions

This analysis suggests that art therapy improves distress in patients with cancer and proposes a simple, reproducible tool to self-report distress, including anxiety, depression, and pain. Art therapy could be used to improve mood, and reduce anxiety and pain levels, among all patients with cancer, who are receiving chemotherapy as noted in our study. These benefits were seen immediately after the session and persisted in follow up, especially in reducing pain and depression. Additionally, this study found that Hispanic patients preferentially benefited from art therapy, despite higher depression scores at baseline. Psychological interventions, including art therapy, are needed to improve the emotional and psychosocial well-being of patients with cancer. The implementation of art therapy, is a cost-effective and culturally appropriate measure, for all cancer patients. Since art-making is universal, this form of therapy may also help overcome language and cultural barriers between patients and their therapeutic care providers, and may facilitate optimal healing of the patient’s mind and body.

## Data Availability

The data that supports the findings of this study may be requested from the Corresponding Author.

## Abbreviations

VAS: Visual analog scale(s)
NCCN: National Comprehensive Cancer Network
REDCap: Research Electronic Data Capture
KS: Kolmogorov-Smirnov
ASCO: American Society of Clinical Oncology
SIO: Society of Integrative Oncology
CES-D: Center for Epidemiologic Study Depression Scale
HADS: Hospital Anxiety Depression scale (HADS)

## References

[1] Strasser F, Sweeney C, Willey J, Benisch-Tolley S, Palmer JL, Bruera E (2004). Impact of a half-day multidisciplinary symptom control and palliative care outpatient clinic in a comprehensive cancer center on recommendations, symptom intensity, and patient satisfaction: a retrospective descriptive study. J Pain Symptom Manage.

[2] Carlson LE, Bultz BD (2004). Efficacy and medical cost offset of psychosocial interventions in cancer care: making the case for economic analyses. Psycho-oncology: journal of the psychological, social and behavioral dimensions of. Cancer..

[3] Holland JC (2003). American Cancer Society award lecture. Psychological care of patients: psycho-oncology's contribution. J Clin Oncol Off J Am Soc Clin Oncol.

[4] Goto E, Hosomi M, Nishihara M, Goto M, Yoshida M, Kii T (2012). Comparison of chemotherapy side effects between elderly and young subjects. Gan To Kagaku Ryoho.

[5] Repetto L (2003). Greater risks of chemotherapy toxicity in elderly patients with cancer. J Support Oncol.

[6] Applebaum AJ, Stein EM, Lord-Bessen J, Pessin H, Rosenfeld B, Breitbart W (2014). Optimism, social support, and mental health outcomes in patients with advanced cancer. Psycho-Oncology..

[7] Waters EA, Liu Y, Schootman M, Jeffe DB (2013). Worry about cancer progression and low perceived social support: implications for quality of life among early-stage breast cancer patients. Ann Behav Med.

[8] Kroenke CH, Kwan ML, Neugut AI, Ergas IJ, Wright JD, Caan BJ (2013). Social networks, social support mechanisms, and quality of life after breast cancer diagnosis. Breast Cancer Res Treat.

[9] Pinquart M, Duberstein PR (2010). Associations of social networks with cancer mortality: a meta-analysis. Crit Rev Oncol Hematol.

[10] Bitonte RA, De Santo M. Art therapy: an underutilized, yet effective tool. Ment Illn. 2014;6(1):5354. 10.4081/mi.2014.5354 PMC4253394 25478139

[11] Lyman GH, Greenlee H, Bohlke K, Bao T, DeMichele AM, Deng GE (2018). Integrative therapies during and after breast cancer treatment: ASCO endorsement of the SIO clinical practice guideline. J Clin Oncol.

[12] Svensk AC, Oster I, Thyme KE, Magnusson E, Sjodin M, Eisemann M (2009). Art therapy improves experienced quality of life among women undergoing treatment for breast cancer: a randomized controlled study. Eur J Cancer Care (Engl).

[13] Kienle GS, Albonico H-U, Baars E, Hamre HJ, Zimmermann P, Kiene H (2013). Anthroposophic medicine: an integrative medical system originating in Europe. Glob Adv Health Med.

[14] Hamre HJ, Witt CM, Kienle GS, Glockmann A, Ziegler R, Rivoir A (2010). Anthroposophic therapy for migraine: a two-year prospective cohort study in routine outpatient settings. Open Neurol J.

[15] Konopka LM (2014). Where art meets neuroscience: a new horizon of art therapy. Croat Med J.

[16] Society AC. Complementary and alternative. Medicine. 2015; Available from: http://www.cancer.org/treatment/treatmentsandsideeffects/complementaryandalternativemedicine/mindbodyandsp.

[17] Bar-Sela G, Atid L, Danos S, Gabay N, Epelbaum R (2007). Art therapy improved depression and influenced fatigue levels in cancer patients on chemotherapy. Psycho-oncology: journal of the psychological, social and behavioral dimensions of. Cancer..

[18] Boehm K, Cramer H, Staroszynski T, Ostermann T. Arts therapies for anxiety, depression, and quality of life in breast cancer patients: a systematic review and meta-analysis. Evid Based Complement Alternat Med. 2014;103297. 10.1155/2014/103297 PMC3955604 24817896

[19] D JGE. ISIS Institute (2010). What is expressive arts therapy 2010.

[20] Mayden KD (2012). Mind-body therapies: evidence and implications in advanced oncology practice. J Adv Pract Oncol.

[21] Wood MJ, Molassiotis A, Payne S (2011). What research evidence is there for the use of art therapy in the management of symptoms in adults with cancer? A systematic review. Psycho-Oncology..

[22] Rubin JA. The art of art therapy: what every art therapist needs to know. Abingdon: Routledge; 2011.

[23] distress Npgftmop (1999). National Comprehensive Cancer Network Oncology (Williston Park). NCCN practice guidelines for the management of psychosocial distress.

[24] Network NCC (2003). Distress management. Clinical practice guidelines. Journal of the National Comprehensive Cancer Network. JNCCN..

[25] Jacobsen PB, Donovan KA, Trask PC, Fleishman SB, Zabora J, Baker F (2005). Screening for psychologic distress in ambulatory cancer patients: a multicenter evaluation of the distress thermometer. Cancer..

[26] Trask P, Paterson A, Riba M, Brines B, Griffith K, Parker P (2002). Assessment of psychological distress in prospective bone marrow transplant patients. Bone Marrow Transplant.

[27] Roth AJ, Kornblith AB, Batel-Copel L, Peabody E, Scher HI, Holland JC (1998). Rapid screening for psychologic distress in men with prostate carcinoma: a pilot study. Cancer: interdisciplinary international journal of the. Am Cancer Soc.

[28] Boyes A, D’Este C, Carey M, Lecathelinais C, Girgis A (2013). How does the distress thermometer compare to the hospital anxiety and depression scale for detecting possible cases of psychological morbidity among cancer survivors?. Support Care Cancer.

[29] Kim EB, Han H-S, Chung JH, Park BR, S-n L, Yim KH (2012). The effectiveness of a self-reporting bedside pain assessment tool for oncology inpatients. J Palliat Med.

[30] Donovan KA, Jacobsen PB (2013). Progress in the implementation of NCCN guidelines for distress management by member institutions. J Natl Compr Canc Netw.

[31] Lynch J, Goodhart F, Saunders Y, O’Connor SJ (2011). Screening for psychological distress in patients with lung cancer: results of a clinical audit evaluating the use of the patient distress thermometer. Support Care Cancer.

[32] Geue K, Goetze H, Buttstaedt M, Kleinert E, Richter D, Singer S (2010). An overview of art therapy interventions for cancer patients and the results of research. Complement Ther Med.

[33] Czamanski-Cohen J, Wiley JF, Sela N, Caspi O, Weihs K (2019). The role of emotional processing in art therapy (REPAT) for breast cancer patients. J Psychosoc Oncol.

[34] Prioli KM, Pizzi LT, Kash KM, Newberg AB, Morlino AM, Matthews MJ (2017). Costs and effectiveness of mindfulness-based art therapy versus standard breast cancer support group for women with cancer. Am Health Drug Benefits.

[35] Monti DA, Kash KM, Kunkel EJ, Moss A, Mathews M, Brainard G (2013). Psychosocial benefits of a novel mindfulness intervention versus standard support in distressed women with breast cancer. Psycho-Oncology..

[36] Monti DA, Kash KM, Kunkel EJ, Brainard G, Wintering N, Moss AS (2012). Changes in cerebral blood flow and anxiety associated with an 8-week mindfulness programme in women with breast cancer. Stress Health.

[37] Meghani SH, Peterson C, Kaiser DH, Rhodes J, Rao H, Chittams J (2018). A pilot study of a mindfulness-based art therapy intervention in outpatients with cancer. Am J Hosp Palliat Med.

[38] Lee J, Choi MY, Kim YB, Sun J, Park EJ, Kim JH (2017). Art therapy based on appreciation of famous paintings and its effect on distress among cancer patients. Qual Life Res.

[39] Koom WS, Choi MY, Lee J, Park EJ, Kim JH, Kim S-H (2016). Art therapy using famous painting appreciation maintains fatigue levels during radiotherapy in cancer patients. Radiat Oncol J.

[40] De Feudis RL, Graziano G, Lanciano T, Garofoli M, Lisi A, Marzano N. An art therapy group intervention for cancer patients to counter distress before chemotherapy. Arts Health. 2019:1–14. (Online ahead of print). 10.1080/17533015.2019.1608566 31044654

[41] Wiswell S, Bell JG, McHale J, Elliott JO, Rath K, Clements A (2019). The effect of art therapy on the quality of life in patients with a gynecologic cancer receiving chemotherapy. Gynecol Oncol.

[42] Kirshbaum MN, Ennis G, Waheed N, Carter F (2017). Art in cancer care: exploring the role of visual art-making programs within an energy restoration framework. Eur J Oncol Nurs.

[43] Forzoni S, Perez M, Martignetti A, Crispino S (2010). Art therapy with cancer patients during chemotherapy sessions: an analysis of the patients' perception of helpfulness. Palliat Support Care.

[44] Archer S, Buxton S, Sheffield D (2015). The effect of creative psychological interventions on psychological outcomes for adult cancer patients: a systematic review of randomised controlled trials. Psycho-Oncology..

[45] Lefèvre C, Ledoux M, Filbet M (2016). Art therapy among palliative cancer patients: aesthetic dimensions and impacts on symptoms. Palliat Support Care.

[46] Quinn K (2014). Mandala art: inter-professional mindfulness education and journaling techniques for self-awareness and self-transformation.

[47] Kim H, Kim S, Choe K, Kim J-S (2018). Effects of mandala art therapy on subjective well-being, resilience, and hope in psychiatric inpatients. Arch Psychiatr Nurs.

[48] Carlson L, Angen M, Cullum J, Goodey E, Koopmans J, Lamont L (2004). High levels of untreated distress and fatigue in cancer patients. Br J Cancer.

[49] Mitchell AJ (2007). Pooled results from 38 analyses of the accuracy of distress thermometer and other ultra-short methods of detecting cancer-related mood disorders. J Clin Oncol.

[50] Zabora J, Brintzenhofeszoc K, Jacobsen P, Curbow B, Piantadosi S, Hooker C (2001). A new psychosocial screening instrument for use with cancer patients. Psychosomatics..

[51] Zigmond AS, Snaith RP (1983). The hospital anxiety and depression scale. Acta Psychiatr Scand.

[52] Gotay CC, Stern JD (1995). Assessment of psychological functioning in cancer patients. J Psychosoc Oncol.

[53] Mitchell AJ, Kaar S, Coggan C, Herdman J (2008). Acceptability of common screening methods used to detect distress and related mood disorders—preferences of cancer specialists and non-specialists. Psycho-oncology: journal of the psychological, social and behavioral dimensions of. Cancer..

[54] Hamilton J, Kruse H, Holcomb L, Freche R (2019). Distress and psychosocial needs: demographic predictors of clinical distress after a diagnosis of cancer. Clin J Oncol Nurs.

[55] González HM, Haan MN, Hinton L (2001). Acculturation and the prevalence of depression in older Mexican Americans: baseline results of the Sacramento area Latino study on aging. J Am Geriatr Soc.

[56] Jang Y, Chiriboga DA, Kim G, Phillips K (2008). Depressive symptoms in four racial and ethnic groups: the survey of older Floridians (SOF). Res Aging.

[57] Rodriguez-Galan MB, Falcón LM (2009). Perceived problems with access to medical care and depression among older Puerto Ricans, Dominicans, other Hispanics, and a comparison group of non-Hispanic whites. J Aging Health.

[58] Russell D, Taylor J (2009). Living alone and depressive symptoms: the influence of gender, physical disability, and social support among Hispanic and non-Hispanic older adults. J Gerontol B Psychol Sci Soc Sci.

[59] Lewis-Fernandez R, Das AK, Alfonso C, Weissman MM, Olfson M (2005). Depression in US Hispanics: diagnostic and management considerations in family practice. J Am Board Fam Pract.

[60] Bureau USC (2007). Statistics on the growth, distribution, and characteristics of the U.S. population.

[61] Bureau USC (2010). Statistics on the growth, distribution, and characteristics of the U.S. population.

[62] Sadule-Rios N, Tappen R, Williams CL, Rosselli M (2014). Older Hispanics' explanatory model of depression. Arch Psychiatr Nurs.

[63] Goetze H, Geue K, Buttstädt M, Singer S, Schwarz R (2009). Art therapy for cancer patients in outpatient care. Psychological distress and coping of the participants. Forschende Komplementarmedizin (2006).

[64] Beck AT, Ward CH, Mendelson M, Mock J, Erbaugh J (1961). An inventory for measuring depression. Arch Gen Psychiatry.

