# Correction to: A pilot study of improved psychological distress with art therapy in patients with cancer undergoing chemotherapy

**DOI:** 10.1186/s12885-020-07643-1

**Published:** 2020-11-20

**Authors:** E. B. Elimimian, L. Elson, E. Stone, R. S. Butler, M. Doll, S. Roshon, C. Kondaki, A. Padgett, Z. A. Nahleh

**Affiliations:** 1 Department of Hematology/Oncology, Maroone Cancer Center, Cleveland Clinic – Florida, 2950 Cleveland Clinic Blvd, Weston, FL 33331 USA; 2 Department of Radiation Oncology, Dana-Farber Cancer Institute/ Brigham and Women’s Hospital, 75 Francis St, Boston, MA 02115 USA; 3 Charles E. Schmidt College of Medicine, Florida Atlantic University, 777 Glades Road BC-71, Boca Raton, FL 33431 USA

**Correction to: BMC Cancer 20, 899 (2020)**

**https://doi.org/10.1186/s12885-020-07380-5**

Following publication of the original article [1], the authors report the following error in the third paragraph of the discussion section. Mandala coloring is not a primary modality used in art therapy, nor was it a primary modality used in the intervention of this study. The first sentence of this paragraph should therefore read “Our data suggests that introspective Mandala drawings, one type of art modality utilized for art therapy, is beneficial.”
